# Large esophageal gastrointestinal stromal tumors resected thoracoscopically after oral imatinib therapy: a report of two cases

**DOI:** 10.1007/s12328-022-01743-0

**Published:** 2022-12-22

**Authors:** Takeshi Kurosaki, Isamu Hoshino, Naoki Kuwayama, Hiroshi Suitou, Masayuki Kano, Toru Tonooka, Satoshi Chiba, Hiroaki Soda, Yoshihiro Nabeya, Wataru Takayama

**Affiliations:** Division of Gastroenterological Surgery, Chiba Cancer Center, 666-2 Nitona-Cho, Chuo-Ku, Chiba, 260-0801 Japan

**Keywords:** Gastrointestinal stromal tumor, Esophagus, Thoracoscopy

## Abstract

Esophageal gastrointestinal stromal tumors (GISTs) are very rare, accounting for 2–5% of all GISTs. As with other GISTs, the principle of surgical treatment is complete resection with negative margins. In addition to biological grades of GISTs itselves, local recurrence due to capsular damage is a known risk. We describe two cases of massive esophageal GISTs that were successfully resected thoracoscopically after 2 months administration of 400 mg imatinib, with some discussion of the literature. Case 1, the patient was a 51-years-old man. After treated with 400 mg of imatinib as preoperative chemotherapy for 2 months, we performed surgery that included right thoracoscopic subtotal esophagectomy, gastric tube reconstruction, and jejunostomy. The resection specimen and histopathology were esophageal GIST-LtMtAeG, 110 × 95 mm. The postoperative course was uneventful, and was discharged on postoperative day 14. The patient has been recurrence free for 11 months postoperatively. Case 2, the patient was a 70-years-old man. After treated with 400 mg of imatinib as preoperative chemotherapy for 2 months, we performed surgery that included right thoracoscopic subtotal esophagectomy, gastric tube reconstruction, and jejunostomy. The resection specimen and histopathology were esophageal GIST-LtAeG, 90 × 52 mm. The postoperative course was uneventful, and was discharged on postoperative day 14. The patient has been recurrence free for 9 months postoperatively.

## Introduction

Gastrointestinal stromal tumors (GISTs) occur in the gastrointestinal tract and mesentery with a prevalence of only 1–2 in 100,000. In particular, esophageal GISTs are very rare, accounting for 2–5% of all GISTs by organ (60–70% for the stomach, 25–35% for the small intestine, 5% for the large intestine, and approximately 2% for the esophagus) [1]. As with other GISTs, the principle of surgical treatment is complete resection with negative margins, and in addition to the biological grade of the GIST itself, local recurrence due to capsular damage is a known risk [2]. In recent years, the Modified Fletcher classification system has been used to classify the risk of GISTs, stratifying risk according to primary site, tumor diameter, and the presence or absence of capsular rupture. Our cases are also classified as a high-risk group due to the fact that it is primary other than the stomach and its diameter exceeds 10 cm. Although, the safety and efficacy of imatinib (400 mg) in high-risk patients with GISTs has been widely agreed for primary gastric GISTs, but no evidence exists for esophageal GISTs. [3]. This is the first report of a successful thoracoscopic resection of a giant esophageal GIST over 10 cm in diameter with neoadjuvant therapy.

## Case reports

### Case 1

**Fig. 1:**
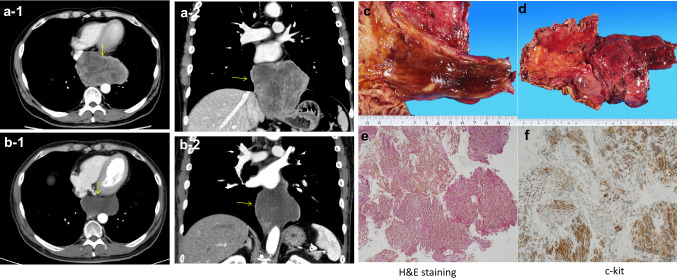
**a** CT showing the tumor (yellow arrow) before neoadjuvant therapy and **b** after neoadjuvant therapy. **c** The mucosal surface of the tumor. **d** The outer surface of the membrane. The tumor diameter was 110 × 95 mm. **e** H&E staining indicated spindle cells proliferation, necrosis and degeneration are also observed in some areas. **f** The tumor cells were positive for c-kit staining

The patient was a 51-years-old man. He visited his previous physician with a chief complaint of dysphagia. He was referred to our hospital for close examination of a large lobulated mass on the left side of the esophagus in the lower chest to the abdomen. His medical history included diabetes and hypertension. He had a life-long history of drinking 1000 ml of beer almost daily and smoking 20 cigarettes per day from the age of 16 until the time of surgery. His blood tests revealed the following: white blood cell count (WBC) 9.3 × 103 K/µl, hemoglobin (Hb) 13.2 g/dl, and tumor markers carcinoembryonic antigen (CEA) 1.2 ng/ml and squamous cell carcinoma (SCC) 0.4 ng/ml, all within normal range. An upper gastrointestinal endoscopy performed at our hospital revealed a 35–45 cm incisor, semi-peripheral submucosal tumor mainly on the left side to the anterior wall, and the lesion partially extended beyond the esophagogastric junction to the gastric side. Endoscopic ultrasound-guided fine-needle aspiration biopsy (EUS-FNA) was performed, and the pathological diagnosis was esophageal GIST. Positron emission tomography/computed tomography (PET–CT) also indicated fluorine-18 deoxyglucose (FDG) accumulation consistent with the same lesion, although no extra-tumoral lymph nodes or other foci were noted. The preoperative diagnosis was a lower thoracic esophageal GIST with a long diameter of 110 mm. Although no remarkable evidence of invasion into the surrounding tissues was observed, the patient was treated with 400 mg of imatinib as preoperative chemotherapy for 2 months to enhance the curative effect. Imaging after medical treatment suggested that the primary tumor had shrunk from 110 to 82 mm, and the patient was advised to undergo radical resection. Surgery included right thoracoscopic subtotal esophagectomy, gastric tube reconstruction, and jejunostomy. First, a right thoracoscopic subtotal esophagectomy of the esophagus was performed at six ports. Since the adhesion to the left pleura, which was in close proximity to the tumor, was strong and the possibility of invasion could not be ruled out, a portion of the pleura was colectomized to avoid exposure of the tumor. Additionally, since part of the tumor was suspected to have invaded the left lung, the lesion was resected in combination with the tumor, and the thoracic operation was completed without damage to the tumor capsule. Surgical manipulation was shifted to the abdomen, which was opened from below the xiphoid process to 2 cm above the umbilicus. The tumor and the right and left diaphragmatic legs were firmly adhered to each other. Both the left and right legs were excised in a combined resection attached to the tumor, and the tumor was pulled out into the abdomen for removal. Subsequently, the patient underwent gastric tube creation, reconstruction via the posterior sternal route, and jejunostomy to complete the surgery. The operative time was 5 h and 26 min, and blood loss was 220 ml. The resection specimen and histopathology were GIST-LtMtAeG, 110 × 95 mm, DOG-1( +), c-kit( +), CD34( +), and fission image was < 1 per 50 fields of view at high magnification. In addition, in the merged resected lung tissue, there was evidence of invasion of septum-like structures leading into the parenchyma. The patient was considered high risk according to the Modified Fletcher classification system. The postoperative course was uneventful, and the patient started oral intake on postoperative day 9 and was discharged on postoperative day 14. He is currently under outpatient follow-up with imatinib 400 mg as postoperative adjuvant chemotherapy, with no evidence of recurrence 11 months after surgery. The patient is planned to continue imatinib for 3 years (Fig. 1).

### Case 2

The patient was a 70-years-old man with no subjective symptoms, but an abnormality was noted during a barium fluoroscopy during a medical checkup. Suspecting a gastric submucosal mass, he visited his previous physician and underwent upper gastrointestinal endoscopy, which revealed an extramural induration near the esophagogastric junction. Contrast-enhanced CT of the thorax and abdomen indicated a mass lesion in the posterior mediastinum, and the patient was referred to our hospital for close examination and treatment. His medical history included hypertension, hyperlipidemia, hyperuricemia, and an enlarged prostate. He had a life history of drinking 1000 ml of shochu 2–3 times a week and smoking 10 cigarettes a day from the age of 20–30 years. Blood tests revealed WBC 4.8 × 103 K/µl, Hb 11.4 g/dl, and tumor markers CEA 3.2 ng/ml and SCC 1.2 ng/ml, all within normal range. EUS-FNA performed at our hospital revealed a mass lesion with extramural induration from the lower esophagus to the gastroesophageal junction, and biopsy results led to a pathological diagnosis of esophageal GIST. PET–CT also suggested FDG accumulation consistent with the same lesion, although no extra-tumoral lymph nodes or other foci were noted. The preoperative diagnosis was esophagogastric junction GIST with a long diameter of 106 mm. Although no remarkable evidence of invasion into the surrounding tissues was noted, the patient was treated with 400 mg of imatinib as preoperative chemotherapy for 2 months to enhance the curative effect.

**Fig. 2:**
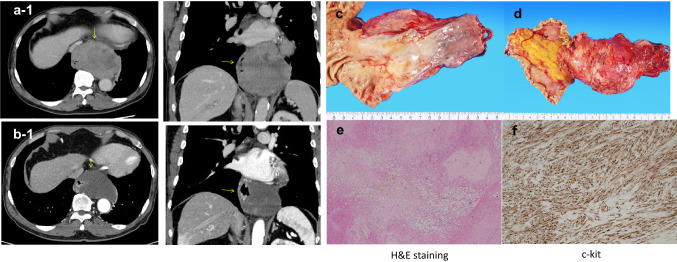
**a** CT showing the tumor (yellow arrow) before neoadjuvant therapy and **b** after neoadjuvant therapy. **c** The mucosal surface of the tumor. **d** The outer surface of the membrane. The tumor diameter was 90 × 52 mm. **e** H&E staining indicated that the tumor cells had no mitotic activity. **f** 30% of the tumor cells were positive for c-kit staining

Imaging after medical treatment indicated that the primary tumor had shrunk from 106 to 90 mm, and since the patient also experienced liver dysfunction as a side effect, the medication was discontinued and the patient underwent a radical resection. Surgery included right thoracoscopic subtotal esophagectomy, gastric tube reconstruction, and jejunostomy. First, a right thoracoscopic subtotal esophagectomy of the esophagus was performed at six ports. The adhesion between the tumor and the left pleura near the tumor was strong, and invasion was suspected. Therefore, a partial pleural resection was performed to avoid exposure of the tumor, and the thoracic operation was completed without damage to the tumor capsule. Surgical manipulation was shifted to the abdomen, and the abdomen was opened from below the xiphoid process to 2 cm above the umbilicus. The tumor and the right and left diaphragmatic legs were firmly adhered to each other. Both the left and right legs were excised in a combined resection attached to the tumor, and the tumor was removed from the abdomen. The patient then underwent gastric tube creation, reconstruction via the posterior sternal route, and jejunostomy to complete the surgery. The operative time was 3 h and 38 min, and blood loss was 35 ml. The resection specimen and histopathology were GIST-LtAeG, 90 × 52 mm, DOG-1( +), c-kit( +), CD34( +),s-100( −), and the fission image was not clear. The patient was considered high risk according to the Modified Fletcher classification system. The postoperative course was uneventful, and the patient started oral intake on postoperative day 9 and was discharged on postoperative day 17. Considering the side effects of preoperative administration of imatinib, the patient is now being followed up as an outpatient without imatinib, and his condition improved without any recurrence 9 months after surgery (Fig. 2).

## Discussion

The safety and efficacy of imatinib (400 mg) as a neoadjuvant therapy before and after surgery in high-risk patients with GIST have been widely reported. However, evidence specifically for esophageal GISTs is lacking. The perioperative recurrence rate of resectable GISTs within 10 years postoperatively is reported to be approximately 20% for intermediate-risk patients and approximately 70% for high-risk patients [4].

The efficacy of preoperative imatinib in resectable GISTs was studied in the RTOG0132 and APOLLON trials, which used 600 mg and 400 mg oral doses, respectively, and no significant benefit was observed with higher doses, with the currently recommended 400 mg/day [5–8]. Additionally, a Japan–Korea phase II trial conducted from 2010 to 2014 was limited to gastric GISTs with a tumor diameter of ≥ 10 cm, and included preoperative imatinib 400 mg/day for 6–9 months and postoperative administration of the same dose for at least 1 year. The primary endpoint of R0 resection was 91% (48/53 patients), and the 2 years overall survival (OS) and 2 years progression-free survival were very good (98% and 89%, respectively) at the median observation period of 32 months [9]. These results demonstrate the benefit of imatinib preoperative adjuvant therapy for large gastric GISTs. Although similar evidence does not exist for esophageal GISTs alone, based on the above results, we introduced 400 mg of imatinib preoperatively to ensure safe and reliable resection in our cases. Consequently, radical resection was achieved with reduction in size. The optimal duration of administration in esophageal GISTs remains unclear, and in our cases, both patients were administered with imatinib for 2 months preoperatively; in the first case, a nearly 30% reduction was achieved, and a radical resection was likely to be performed based on imaging. In the second case, the reduction rate was not large, although the possibility of radical resection was considered high, and liver dysfunction due to imatinib would make it difficult to continue treatment. Many locally advanced large GISTs are fragile and hypervascularized and can easily rupture or sustain capsular injury intraoperatively with the risk of subsequent dissemination. Neoadjuvant therapy may reduce the extent of resection due to tumor shrinkage, the risk of capsular injury, and the amount of bleeding due to the reduction of tumor vessels, thereby reducing surgical invasiveness and postoperative complications.

Two surgical approaches have been reported: open chest/abdomen or endoscopic surgery. In endoscopic surgery, forceps movement is restricted and securing a working space may be difficult, especially for lesions with a large tumor diameter. However, the possibility of avoiding capsular damage through precise surgery with the magnification effect unique to the specular approach and the reduction of chest wall destruction compared to open thoracotomy can be expected to reduce surgical invasiveness and pain, as well as the risk of postoperative respiratory complications. On the other hand, thoracoscopic surgery in cases of GIST with large tumor diameter requires special attention to capsular damage associated with mobility limitation. In our case, the tumor was large in diameter and in close proximity to major blood vessels and surrounding organs, although complete resection could be performed without capsular damage. To date, only one case of thoracoscopic resection with neoadjuvant therapy has been reported. (Table 1).

**Table 1:** Reported cases of resection for esophageal GISTs with a tumor diameter of > 100 mm

Case no	Age, sex	Tumor distance from incisor (cm)	Tumor size(mm)	Neoadjuvant chemotherapy	Operative procedure	References
1	59, male	35–40	135	None	Ivor Lewis esophagectomy	Gouveia et al. [10]
2	75, male	38	140	None	Left thoracoabdominal gastroesophagectomy	Manu et al. [11]
3	33, female	33	270	No date	Proximal gastrectomy, subtotal esophagectomy, and esophagogastrostomy	Basoglu et al. [12]
4	74, female	26–36	125	No date	Right thoracotomy enucleation with excision of surrounding muscle	Blum et al. [13]
5	50, female	30–36	109	Imatinib 400 mg × 6 months	Esophagogastrectomy along with distal pancreaticospleenectomy	Krishnamurthy [14]
6	39, male	No date	190	None	Left thoracoabdominal gastroesophagectomy	Sjogren et al. [15]
7	29, male	24–34	130	None	Enuclearation surgery through the right posterolateral thoracotomy	Mu et al. [16]
8	49, male	No date	180	Imatinib 400 mg × 4 months	Esophagectomy via left thoracotomy	Takeno et al. [17]
9	65, female	32–42	180	None	Esophagectomy via left thoracotomy and laparotomy	Nakano et al. [18]
10	50, female	No date	104	Imatinib 400 mg × 10 months	Laparoscopic-assisted Ivor Lewis esophagectomy	Neofytou et al. [19]
11	51, male	35–45	110	Imatinib 400 mg × 2 months	Right thoracoscopic subtotal esophagectomy/poststernal route gastric tube reconstruction	Our case 1
12	70, male	No date	106	Imatinib 400 mg × 2 months	Right thoracoscopic subtotal esophagectomy/poststernal route gastric tube reconstruction	Our case 2

A retrospective, multicenter study of esophageal GIST cases from 2000 to 2019 in seven European countries (Netherlands, Poland, Germany, Belgium, United Kingdom, Italy, and Spain) has reported that tumor size is related to the risk of disease progression and that surgical resection is recommended for patients with tumors of > 4 cm in diameter [20].

Regarding postoperative adjuvant therapy, the Z9001 study [21, 22], EORTC62024 study [23], and SSGXVII/AIO study [24] have reported that 400 mg of imatinib for 3 years was beneficial for recurrence-free survival (RFS) and OS in high-risk patients with GISTs for whom neoadjuvant therapy was considered. The SSGXVIII/AIO study was a phase III trial conducted in Europe comparing 1 year versus 3 years postoperative treatment with imatinib 400 mg/day. Long-term follow-up results also indicated a significant difference in 5 years OS (91.9% in the 3 years group and 85.3% in the 1 year group) (*p* = 0.036) [25]. However, it should be noted that in the SSGXVIII/AIO study, only 83% of patients underwent R0 resection, and also included cases of tumor rupture, which is debatable. Here, we refrained from administering postoperative adjuvant chemotherapy in one case due to side effects during preoperative administration, although aggressive administration is desirable in patients who can receive oral therapy. All procedures were in accordance with the ethical standards of the responsible committee on human experimentation and with the Helsinki Declaration of 1964 and its later amendments.

## Conclusion

We successfully performed thoracoscopic resection after providing oral imatinib mesylate in two cases of massive esophageal GIST. Thoracoscopic esophagectomy may be considered as a treatment option for esophageal GISTs that are judged to be curative. Preoperative imatinib mesylate 400 mg may be useful in massive esophageal GISTs. Thoracoscopic esophagectomy is also considered a treatment option for esophageal GISTs that are considered curable.

## Data Availability

Data sharing is not applicable to this article, as no datasets were generated or analyzed during the study.
